# Mechanisms of Endothelial Dysfunction in Pre-eclampsia and Gestational Diabetes Mellitus: Windows Into Future Cardiometabolic Health?

**DOI:** 10.3389/fendo.2020.00655

**Published:** 2020-09-11

**Authors:** Colm J. McElwain, Eszter Tuboly, Fergus P. McCarthy, Cathal M. McCarthy

**Affiliations:** ^1^Department of Pharmacology and Therapeutics, Western Gateway Building, University College Cork, Cork, Ireland; ^2^Department of Obstetrics and Gynaecology, Cork University Maternity Hospital, Cork, Ireland

**Keywords:** PE, GDM, mitochondria, oxidative stress, adiposity, endothelial dysfunction, therapeutics

## Abstract

Placental insufficiency and adipose tissue dysregulation are postulated to play key roles in the pathophysiology of both pre-eclampsia (PE) and gestational diabetes mellitus (GDM). A dysfunctional release of deleterious signaling motifs can offset an increase in circulating oxidative stressors, pro-inflammatory factors and various cytokines. It has been previously postulated that endothelial dysfunction, instigated by signaling from endocrine organs such as the placenta and adipose tissue, may be a key mediator of the vasculopathy that is evident in both adverse obstetric complications. These signaling pathways also have significant effects on long term maternal cardiometabolic health outcomes, specifically cardiovascular disease, hypertension, and type II diabetes. Recent studies have noted that both PE and GDM are strongly associated with lower maternal flow-mediated dilation, however the exact pathways which link endothelial dysfunction to clinical outcomes in these complications remains in question. The current diagnostic regimen for both PE and GDM lacks specificity and consistency in relation to clinical guidelines. Furthermore, current therapeutic options rely largely on clinical symptom control such as antihypertensives and insulin therapy, rather than that of early intervention or prophylaxis. A better understanding of the pathogenic origin of these obstetric complications will allow for more targeted therapeutic interventions. In this review we will explore the complex signaling relationship between the placenta and adipose tissue in PE and GDM and investigate how these intricate pathways affect maternal endothelial function and, hence, play a role in acute pathophysiology and the development of future chronic maternal health outcomes.

## Introduction

Gestational diabetes mellitus (GDM) and pre-eclampsia (PE) are two obstetric complications which detrimentally impact perinatal outcomes for both mother and child. For the mother, PE has been associated with chronic conditions of endothelial damage and cardiovascular events (1). GDM has also been linked to long-term alteration of maternal endothelial function and a greater risk of type 2 diabetes mellitus (T2DM) diagnosis (2). A shift in glucose metabolism, during which maternal hyperinsulinemia is compensated to support the need of fetal growth, serves as a cardiometabolic stressor that may culminate in CVD co-morbidities or long-term cardiovascular complications (3).

Similar risks exist for the offspring, such as neurodevelopmental disorders in hypertensive pregnancies and type 1 diabetes mellitus in hyperglycemic pregnancies such as GDM (4, 5). Population based studies have shown that the deleterious implications of obstetric complications on the fetus, such as an increased risk of endocrine, nutritional, and metabolic derangements, including T2DM, are present well into adolescence and early-adulthood (6, 7).

GDM is the most common metabolic pregnancy complication and affects up to 18% of all pregnancies, however this value differs greatly (between 14 and 25%) depending on the diagnostic test and glucose cut-off values employed (8). During healthy pregnancy, the maternal physiology adapts to compensate for many changes in energy demands, such as glucose metabolism. As the fetus develops, a surge of hormone secretion, such as placental lactogen and estrogen, promote a state of insulin resistance, which facilitates a rise in blood glucose levels to fuel fetal growth (9). Appropriate blood glucose levels are then maintained through homeostatic mechanisms such as hypertrophy and hyperplasia of pancreatic β-cells (10). However, in GDM pregnancies, normal metabolic adaptions fail to adequately control glycemic levels, resulting in hyperglycemia and secondary complications such as vasculopathy and birthing complications (11). GDM outcomes have been linked to issues upstream of dietary glucose absorption, such as impaired insulin sensitivity caused by β-cell dysfunction (12). The International Association of Diabetes and Pregnancy Study Groups (IADPSG) recently updated the diagnostic criteria for GDM whereby an oral glucose tolerance test (OGTT) is performed in a fasting state using 75 g of glucose at 24–28 weeks. GDM is positively diagnosed if any one of the following cut-off's is met i.e., ≥ 92 mg/dl (≥5.2 mmol/l) or 1 h ≥ 180 mg/dl (≥10 mmol/l) or 2 h ≥ 153 mg/dl (≥8.5 mmol/l) (13).

PE affects 2–8% of pregnancies and has large heterogeneity in its associated risk factors (14, 15). The exact pathophysiology of PE remains a controversial issue, however it is well-accepted that complications originate during abnormal placentation and resulting placental insufficiency. During normal placentation, cytotrophoblast cells facilitate remodeling of the maternal uterine spiral arteries, allowing for normal blood flow and nutrient supply to the growing fetus (16). The fusion of cytotrophoblast cells to terminally differentiated syncytiotrophoblasts, allowing for the formation of gap junctions and the exchange of small metabolites and secondary messengers, is a complex event which is not fully understood (17). In cases of PE, particularly early onset PE (delivery <34 weeks), trophoblast abnormalities has been postulated as culprits of poor placentation and atherosclerotic changes in the placental vasculature (18). The resulting hypoxic environment and localized oxidative stress responses are believed to offset systemic responses, such as hypertension, proteinuria and HELLP syndrome, which define PE (16). Symptoms of late onset PE (delivery ≥34 weeks) are likely to occur due to abnormal placental senescence and a maternal genetic predisposition to cardiovascular and metabolic disease (18). Contrary to previous criteria, recent guidelines from the International Society for the study of Hypertension in Pregnancy (ISSHP) have outlined that proteinuria is not mandatory for a diagnosis of PE. Rather, this is diagnosed by the presence of *de novo* hypertension after 20 weeks' gestation accompanied by proteinuria and/or evidence of maternal acute kidney injury (AKI), liver dysfunction, neurological features, hemolysis or thrombocytopenia, or fetal growth restriction (19).

The pathophysiology of PE and GDM has complex implications on maternal health, which include the possibility of unmasking existing CVD predispositions. However, both complications have established risk factors which can be identified pre-pregnancy or early in pregnancy and efforts to promote early screening and risk-reduction strategies may significantly improve health outcomes. Some risk factors common to both conditions include maternal obesity, pregestational diabetes, maternal age, previous PE and/or GDM diagnosis, and familial history (9, 20–23). Due to the increase in obesity levels and Western dietary practices, GDM diagnoses have dramatically increased over the past decade (24). While recent reports have suggested that PE rates have remained static, the number of severe cases are on the rise (25). Examining clinical risk factors allow for some diagnostic predictions, however, this is not a sufficient method to decide the need for therapeutic intervention such as prophylactic aspirin in patients of high PE risk (26). Further elucidation of the pathophysiology of these obstetric conditions will provide a clearer insight into the most effective means for clinical diagnosis and therapy. In this review we will describe how deterioration of the endothelial vasculature as a consequence of these obstetric complications may be implicated in long-term maternal cardiometabolic disease. We will aim to delineate potential pathogenic mediators of endothelial dysfunction and discuss potential therapeutic targets that may ameliorate this disruptive pathophysiology.

## Does PE and GDM-Mediated Endothelial Dysfunction Contribute to The Future Risk of Maternal Cardiometabolic Disease?

It has been previously postulated that endothelial dysfunction plays a pivotal role in the detrimental health outcomes recorded in PE and GDM, playing a particular role in hypertension, proteinuria, cardiovascular risk, obesity and hyperlipidemia (27). Recent studies have shown that women diagnosed with PE and GDM have a significantly lower flow-mediated dilation (FMD) compared with their healthy counterparts, suggestive of increased endothelial damage in these patients (1, 28). Persistent endothelial dysfunction in these patients may lead to maternal cardiovascular disease (CVD) and poor cardiometabolic health, dominant causes of mortality globally and major factors of concern in maternal health both pre- and post-partum. Cardiometabolic disease encapsulates a spectrum of conditions, which develop from insulin resistance and visceral obesity into a decrease in high-density lipoprotein (HDL) cholesterol concentrations, an increase in triglyceride concentrations, and hypertension. These metabolic factors manifest as serious clinical outcomes such as CVD and type II diabetes (29). Additionally, PE and GDM are, themselves, established risk factors for future poor cardiovascular health outcomes (30). Although the acute symptoms of these obstetric complications are alleviated post-delivery, the occurrence of these is associated with a predisposition or vulnerability for the pregnant woman to develop longer term chronic maternal complications which may not be clinically detectable for years after pregnancy. For example, the risk of developing vascular complications later in life is increased up to 14.5-fold in women after hypertensive pregnancies (31).

One probable theory for postpartum CVD correlation is the persistence of endothelial dysfunction. For example, in PE pathophysiology, inadequate cytotrophoblast invasion and resultant insufficient placentation causes a decrease in placental blood flow due to poor spiral artery remodeling. A compensatory rise in maternal blood pressure and oxidative stress provokes pro-inflammatory signaling pathways with the resultant generation of cytokines which induce endothelial dysfunction, thereby causing increased arterial lipid deposition and arterial stiffness (32).

Recent evidence has also suggested that the maternal vasculature is permanently disrupted in GDM, which predisposes the mother to a higher risk of developing CVD, including an increased risk of ischemic heart disease, myocardial infarction, coronary angioplasty, and coronary artery bypass graft (33). This risk is due, in part, to an increase in T2DM or intrinsic endothelial dysfunction due to intense glucose intolerance (34, 35). A recent US study established a 63% increased risk of CVD amongst women with a history of GDM, which was only partly explained by correlation with body mass index (BMI) (36), suggesting other key players in GDM pathophysiology. Maternal comorbidities such as obesity and dyslipidemia may have further deleterious effects on maternal endothelial function. There is also increasing evidence that these cardiometabolic conditions may be dormant predispositions, whether genetic or otherwise, which are unmasked by pregnancy complications.

**Figure 1:**
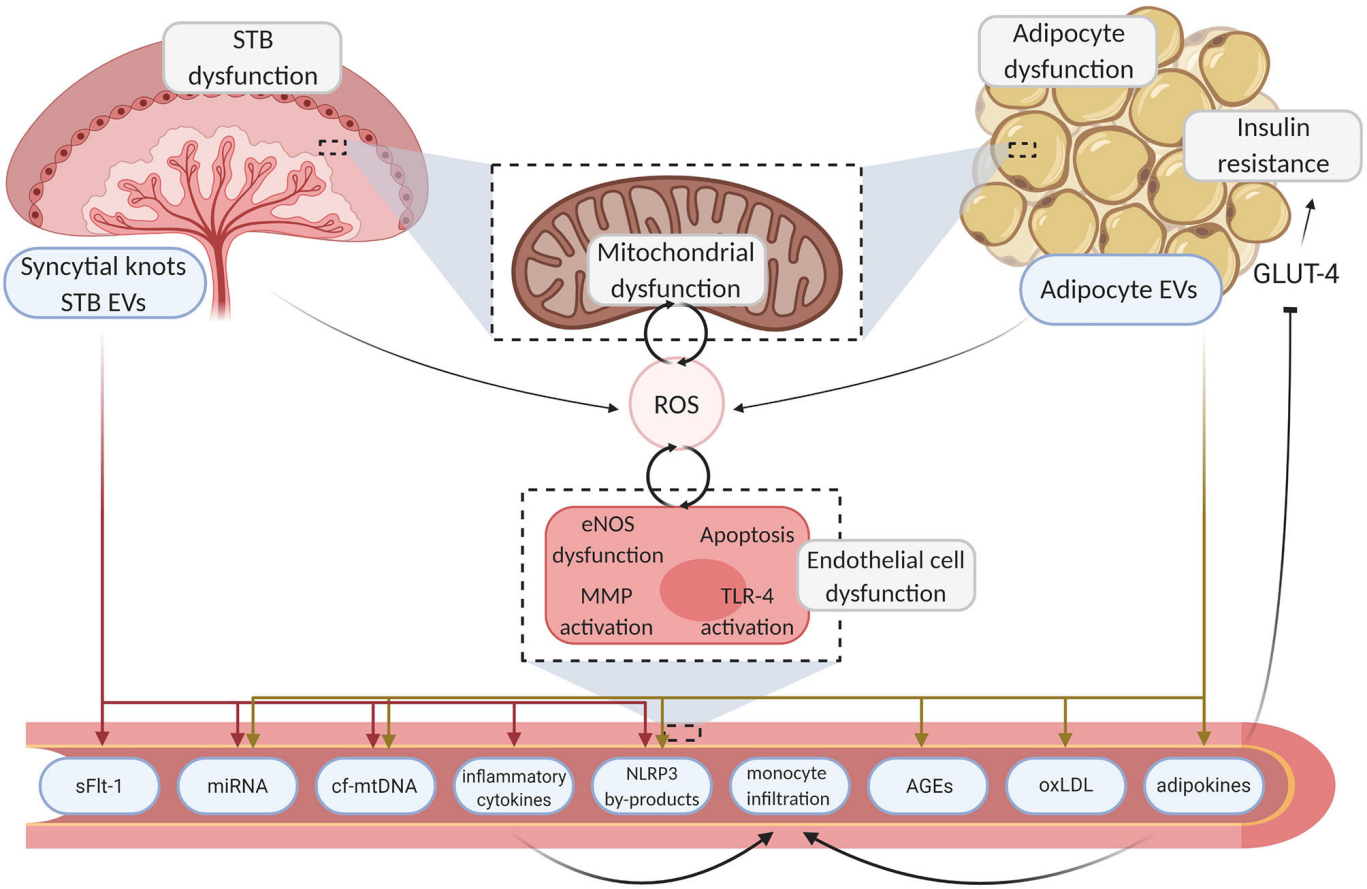
Mechanisms linking placenta and adipose tissue dysfunction to endothelial damage. Aberrant endocrine organ dysfunction leads to excessive ROS generation and the release of inflammatory motifs from the placenta and adipose tissue. Deleterious motifs such as NLRP3 by-products, miRNA, inflammatory cytokines, and mtDNA enter the circulation, instigating various pathways which drive localized damage to the endothelium through inhibition of angiogenesis and inflammation. Sequential endothelial dysfunction ensues and pathogenic pathways such as TLR-4 driven cytokine production further upregulate ROS generation, creating a pernicious feedback loop. Maternal vasculopathy ensues, resulting in acute complications and poor chronic health outcomes. Created with BioRender.com. STB, syncytiotrophoblast; EV, extracellular vesicle; ROS, reactive oxygen species; GLUT-4, glucose transporter type 4; eNOS, endothelial nitric oxide synthase; MMP, matrix metallopeptidase; TLR-4, toll-like receptor 4; sFLT-1, soluble fms-like tyrosine kinase-1; miRNA, micro ribonucleic acid; cf-mtDNA, cell-free mitochondrial deoxyribonucleic acid; NLRP3, NOD-; LRR- and pyrin domain-containing protein 3; AGE, advanced glycation endproducts; oxLDL, oxidized low-density lipoprotein.

Two endocrine organs which undoubtedly play a pathophysiological role in both PE and GDM are the placenta and adipose tissue. Both metabolic organs release a myriad of signals which can induce deleterious effects on the maternal vasculature, with probable long-term consequences. Potential mediators which have been proposed in directing the deleterious communication between both metabolic organs and the maternal endothelium include reactive oxygen species (ROS), mitochondrial DNA, pro-inflammatory cytokines and lipid-derived signals (Figure 1). In this review we will explore the intertwined signaling relationships of placenta and adipose tissue on maternal endothelial function and investigate how these mediators play a pathophysiological role in future maternal cardiometabolic outcomes of pregnancies with PE and GDM through disruption of endothelial function.

## Pathogenic Contributors to Endothelial Damage

### Reactive Oxygen Species

Physiologically, pregnancy is associated with oxidative stress, largely orchestrated by placental mitochondrial activity and the production of reactive oxygen species (ROS), by-products of normal cellular activity (37). Mitochondria serve as the primary source of endogenous ROS, however the endoplasmic reticulum and peroxisomes also function in ROS production (38). Excessive ROS generation can lead to the release of deleterious mediators into the maternal circulation and this overproduction is overtly evident in insufficient placentation and the resulting ischemic microenvironment in the placenta (39).

Although certain immune cells such as uterine natural killer cells (uNK) and macrophages prime the smooth muscle and endothelium for invasion, specifically the extravillous cytotrophoblast (EVT) plays a major role in vascular infiltration of the decidua and myometrium (40). Placental insufficiency, a condition which has been postulated as a culprit in obstetric complications including PE and intrauterine growth restriction (IUGR), results from insufficient or incomplete trophoblast invasion of the maternal uterine spiral arteries (41, 42). The resulting impaired uteroplacental blood flow leads to diminished placentation, ischemia, oxidative stress, inflammation and apoptosis of the syncytiotrophoblast (43, 44). Similar pathological pathways are activated in cases of maternal obesity, where increasing visceral adipose tissue mass increases adipocyte dysfunction, resulting in inflated ROS production. This hyperbolic ROS generation has been correlated to an increase in insulin resistance in both the adipose and other peripheral tissues (45).

Excessive ROS production is postulated to play a significant role in mediating many vascular responses, specifically activation of matrix metalloproteinases (MMPs), vascular remodeling, smooth muscle hypertrophy and cellular apoptosis. In response to ROS, oxidation of the IκB kinase (IKK) complex occurs, leading to the release of nuclear factor kappa B (NF-κB) (46) which promotes the transcription of various pro-inflammatory mediators of endothelial dysfunction including intracellular adhesion molecule 1 (ICAM-1), the vascular cell adhesion molecule 1 (VCAM-1) and inflammatory cytokines such as interleukin (IL)-6 and tumor necrosis factor (TNF)-α (47). This interplay is appropriately regulated during pregnancy but becomes disorientated in PE and GDM (48, 49).

Exaggerated ROS generation thereby represents a primary cause of endothelial dysfunction in both PE and GDM, resulting in potentially permanent vascular damage and altered endothelial phenotype which may have significant long term consequences (50).

### Nitric Oxide

Pregnancy is associated with significant physiologic adaptive changes of the maternal cardiovascular system. Nitric oxide (NO), a soluble gaseous mediator, has a wide variety of physiological functions including maintenance of vascular homeostasis and modulating vascular tone (51). ROS can interfere with the maintenance of vascular tone through reduction of nitric oxide (NO) production. Endothelial NO synthase (eNOS), which is expressed constitutively in the vascular endothelium and regulates vascular tone through NO synthesis, appears to be suppressed by ROS overproduction (52). Suppression of endothelial NO synthesis causes dysregulation in vascular tone modulation, and platelet and leukocyte adhesion (51).

NO malfunction has been specifically implicated in PE and GDM-derived endothelial complications through abnormal maternal vascular adaption mechanisms (53, 54). During pregnancy, NO produced by endothelial and inducible NO synthases (eNOS and iNOS, respectively) actively regulates embryo development, implantation, trophoblast invasion, placental vascular development and function (40, 41). There has been inconclusive evidence on the levels of NO expression throughout gestation, predominantly due to the variances in the NOS levels recorded in maternal serum (55, 56).

eNOS and the concurrent synthesis of NO has been implicated in maternal endothelial dysfunction, however the exact pathophysiology of this mechanism remains elusive. A recent study reported that women with severe hypertensive pregnancies had significantly lower circulating eNOS levels and that this was significantly associated with a decrease in levels of placental growth factor (PlGF), while a subtle increase in eNOS and PIGF was recorded in women with mild hypertensive pregnancies (57). These findings may suggest that although a compensatory rise in eNOS and PlGF levels exists in mild pathologies, these mechanisms fail in severe hypertensive cases. Both eNOS and iNOS have been shown to be tightly regulated in placental tissue throughout pregnancy, and a number of studies have reported on evidence of a dysregulated increase in eNOS response with conditions of placental hypoxia such as that which is seen in placental insufficiency and impaired vascular development (58–60). This induces a compensatory increase in NO-induced vasodilation of the placental vasculature. Additionally, further work in this area has established that the quantity of syncytiotrophoblast extracellular vesicles (EVs) carrying eNOS was reduced in PE patients relative to control patients (61). This observed reduction in circulating eNOS may correlate to a reduction in NO bioavailability and an increase in endothelial dysfunction.

Although the direct interaction between NO and ROS regulates the maintenance of physiological vascular tone during normal pregnancy (62), their imbalance may also contribute to pathogenic effects. In a recent clinical study of women with GDM, there was evidence of increased eNOS expression in both maternal and cord blood, compared to matched healthy controls, but a decrease in circulating NO levels (2). This phenomenon, known as eNOS uncoupling, occurs due to increased monomerization of the eNOS enzyme when the eNOS dimer is disrupted due to the presence of peroxynitrite, a by-product of the scavenging of NO by the superoxide radical (O_2_-) (63). The resulting damage leads to superoxide production by eNOS rather than the generation of NO (63). This production of superoxide, in addition to excess ROS produced from other sources such as endothelial cell mitochondria under conditions of hypoxia and hyperglycemia, mediates inactivation and sequestration of NO through oxidative reactions, contributing to hypertension and tissue damage (64, 65).

Endothelium-derived NO dysfunction, resulting in altered bioavailability and tissue damage, has been specifically implicated as a potential cause of PE symptoms (66) and, similar to above, the interplay between ROS and NO plays a key role. A predominant source of ROS is due to the disrupted membrane potential of endothelial cell mitochondria. This occurs in response to mechanical damage to the mitochondria or in response to oxidative stress, often initiating ROS overproduction, particularly at complexes I and III. Another localized source of ROS is nicotinamide adenine dinucleotide phosphate (NADPH) oxidase 4 (Nox4), which is highly expressed in endothelial cells and is essential for cellular proliferation (65). Nox4 is upregulated in cases of cellular stress, particularly in those with impaired blood flow or hyperglycemia (67). Once levels of ROS exceed cellular buffering capacity, dysfunction and apoptosis ensue. Peroxynitrite, produced through ROS scavenging of NO, oxidizes DNA, proteins and lipids and contributes to vascular signaling dysregulation (52). Furthermore, peroxynitrite can also contribute to irreversible nitration of tyrosine residues on other proteins, causing impaired phosphorylation and enzymatic dysfunction (68). Thus, this interaction between NO and ROS propagates endothelial damage and may be another key element in PE and GDM-mediated vasculopathy.

### Mitochondrial Dysfunction

As described earlier, mitochondria are the primary mediators of oxidative stress and consequently influence exaggerated ROS mediated endothelial dysfunction (65). However, an additional mechanism by which mitochondria can disrupt endothelial function is mediated in part by mitochondrial DNA (mtDNA) provocation of an inflammatory response. Mitochondria have their own genome with mtDNA molecules, which are particularly susceptible to oxidative damage because of their proximity to the electron transport chain (ETC) and their deficiency of protective histones (69). A change in membrane potential, brought on by traumas such as oxidative stress and pathogen invasion, induces mitochondrial membrane depolarization and increases permeability (70). These changes facilitate the release of mitochondrial components such as ROS and mtDNA into the cytosol, triggering various inflammatory and apoptotic pathways (71). Damage to mtDNA can induce malfunction of the electron transport chain and adenosine triphosphate (ATP) production, resulting in ROS overproduction by complex I and III (72). The above pathway describes a pernicious feedback loop, where mtDNA can be damaged by ROS-mediated oxidation leading to diminished oxidative phosphorylation capacity, mtDNA fragment release and further ROS production (73).

Secreted mtDNA fragments also act as damage-associated molecular patterns (DAMPs), endogenous signaling molecules that are released from damaged cells and activate an innate immune response (74). MtDNA can instigate a pro-inflammatory response in the maternal circulation by binding to toll-like receptor 9 (TLR-9) on immune cells. Recent work in our group established that pathogenic plasma mediators (including mtDNA) of PE increased TLR-9 activation with ensuing neutrophil activation (75, 76). MtDNA has also been shown to activate the NLRP3 inflammasome leading to cleavage of procaspase-1 and the resulting release of pro-inflammatory cytokines IL-1β and IL-18, which in turn can inflict endothelial damage (77, 78). Persistent and aberrant NLRP3 activation underlies many chronic inflammatory and degenerative diseases, such as T2DM and CVD (77, 79–81).

Previous studies have suggested that cell-free circulating mtDNA (cf-mtDNA) copy number increases with mitochondrial dysfunction (82). Although the majority of cf-mtDNA in the maternal circulation is of maternal origin, ~5–20% of this cf-mtDNA is of fetal/placental origin (83). Our group have shown elevated levels of circulating mtDNA fragments in plasma samples from 15 to 20 weeks' gestation from both PE and GDM patients (69, 84). An additional source of cf-mtDNA in the maternal circulation could be from adipose tissue, as mitochondrial dysfunction in the omental adipose tissue has been linked to a significant increase in mtDNA content (85). Adipocyte mitochondria have been shown to play a key role in signaling through their ability to influence key biochemical processes central to the adipocyte, such as fatty acid esterification and lipogenesis, as well as their impact on production and release of key adipokines (86).

Mitochondria are also primary apoptotic mediators (87). Endogenous cellular stressors such as mtDNA and ROS can activate BH3-only proteins, which inhibit anti-apoptotic factors, stimulate pro-apoptotic protein synthesis and lead to further mitochondrial damage through outer membrane permeabilization (88, 89). The resulting release of cytochrome C and other mitochondrial proteins activates pro-inflammatory caspases such as the NLRP3-caspase-1 pathway (87). Oxidized low-density lipoproteins (oxLDL), which are also released from damaged mitochondria following membrane permeabilization, have been directly linked to endothelial cell apoptosis through ROS upregulation and p53 activation, a tumor supressing gene which induces cell growth arrest and apoptosis (89, 90). It has also been postulated that nitration of mitochondrial protein complex I and membrane depolarization in endothelial cells contributes to cellular necroptosis, through alteration and inhibition of mitochondrial bioenergetics (91).

Brown adipose tissue (BAT) is distinct from white adipose tissue (WAT) in that their more abundant mitochondria profoundly express uncoupling protein 1 (UCP1), which uncouples substrate oxidation from ATP production so that heat can be produced (92). Decreased secretion of the adipokine neuregulin 4 (NRG-4) by BAT was recently described in 74 women with GDM (93). NRG-4 is a novel member of the neuregulin family which is predominantly secreted by BAT and has been suggested to play a vital role in regulating the mitochondrial oxidative machinery (94). Another recent study reported a direct inter-tissue communication between BAT and the placenta, via placental growth factor (PIGF), which manifests in increased UCP-1 expression and mitochondrial oxygen consumption in GDM BAT tissue when compared to normal pregnancy (95). The importance of the metabolic mitochondria in influencing endothelial function cannot be underestimated, whether its directly via ROS-mediated oxidative stress or indirectly via instigation of pro-inflammatory signaling pathways.

### Pro-inflammatory Cytokines

The placenta, an important endocrine organ, is also a key instigator of inflammatory signaling. Placental tissue is largely maintained by successful syncytiotrophoblast functionality (96). A release of pro-inflammatory motifs is a key stress response of the syncytiotrophoblast, which contributes largely to a localized oxidative stress response in the placenta, leading to an increased secretion of antiangiogenic and pro-inflammatory messengers, thereby significantly influencing maternal response and endothelial impairment (97).

Cytokines are small secreted proteins which regulate the functions of the immune system, having specific effects on communications between cells (98). In healthy uncomplicated pregnancy, complex cytokine signaling in the placenta mediates local vascular growth and development. Tissue-specific expression of cytokines facilitates additional functions during gestation, for example, IL-8 expression in the myometrium has been shown to be specifically upregulated during both term and preterm labor (99). However, dysregulation of placental cytokine release has been demonstrated to correlate with obstetric pathology. In pre-eclamptic mothers, higher levels of TNF-α and transforming growth factor (TGF)-β1 have been noted in placental histopathological studies, whereas level of IL-10, regarded as a potent anti-inflammatory cytokine, were decreased in PE vs. normotensive placentas (100). A correlated “cytokine signature” has been described in the maternal circulation, with significantly higher levels of pro-inflammatory cytokines IL-8, IL-6, and IFN-γ in PE plasma samples, relative to normotensive women. These findings were particularly exaggerated in cases of severe PE (101). Similar research demonstrated that human uterine decidual cell-derived IL-6 was found to contribute to excess circulating IL-6 levels that promote both endothelial cell dysfunction (and subsequent vascular dysfunction) in the pathogenesis of PE (102).

Also elevated in PE, anti-angiogenic soluble fms-like tyrosine kinase-1 (sFlt-1) and soluble endoglin (sENG) levels, released in an exacerbated inflammatory states, are culprits for endothelial dysfunction, whereas proangiogenic PlGF levels, which are involved in preserving endothelial function, are reduced in pathologies of placental dysfunction (103).

Adipose tissue is also a highly metabolic endocrine organ which has a substantial impact on signaling mediators, including those from the placenta, which instigate a biological response in the mother and, as such, have considerable implications for maternal cardiometabolic health (104). Adipose tissue secretes various humoral factors, known as adipokines, which can regulate both pro- and anti-inflammatory responses. This is particularly evident in the metabolically active visceral adipose tissue. Furthermore, immune cells including monocytes, macrophages, dendritic cells, natural killer (NK) cells, mast cells and granulocytes have been shown to infiltrate visceral adipose tissue and stimulate metainflammation (via increased production of pro-inflammatory cytokines) and adipocyte dysregulation (105). TLR-4 activation in adipocytes initiates adipose inflammation and a systemic innate immune response (106). Triggering of TLR-4 receptor activation is thought to be regulated by free fatty acids (FFAs), which is linked to an increase in obesity and lipolysis (106). Dysregulation of adipokine secretion and adipogenesis are considered risk factors and symptoms of PE and GDM (107, 108).

Some of the key adipose-derived factors involved are leptin, adiponectin, resistin, TNF-α, IL-6, and IL-1β (109). Other than adiponectin, which can act in both an anti- and pro-inflammatory capacity (110), these adipokines predominantly exert a pro-inflammatory response. Adipokines have been also shown to regulate vascular function, often resulting in vascular inflammation with consequent development of atherosclerosis (104). The deleterious impact that adipokines have on the endothelium and vascular homeostasis is complex. For example, leptin, an adipokine implicated in energy intake and expenditure, binds to receptors of the endothelial surface and regulates vascular tone by phosphorylating mitogen activated protein (MAP) kinases (111). Further to this, overproduction of leptin has been linked to decreased NO bioavailability through an increase in oxidative stress (112). Leptin also plays a role in platelet activation and aggregation, contributing to thrombosis and CVD (113).

Maternal adiposity has also been shown to disrupt placental-derived cytokine signaling. Challier et al. reported that obesity in pregnant women resulted in a 2–3-fold increase in the number of placental macrophages, characterized by an increase in IL-1, TNF-α, and IL-6 mRNA expression (114). Maternal obesity has also been characterized by an increase in placental expression of both IL-6 and TNF-α, while similar studies have found an increase in IL-8 and leptin expression in the placentas of GDM women (115). Hofbauer cells (HBCs), placental-specific macrophages, have been implicated in regulating placental inflammatory signaling in both obstetric complications. The physiology of this pathway is thought to involve a switch from the anti-inflammatory M2 phenotype to the pro-inflammatory M1 phenotype. Sisino et al. supported this hypothesis by showing that, in a rodent model, gestational diabetes altered the normal phenotype of HBCs from the M2 to the M1 subtype (116). On the contrary, analysis of isolated HBCs showed an increase in M2 markers such as CD206 and CD206 in the placentae of GDM women. Although not significant, an increase in the secretion of IL-1β and IL-6 from GDM HBCs was also recorded (117), indicating that more investigation is needed to establish the bidirectional effects of HBCs and obstetric pathology.

Other pro-inflammatory mediators including resistin, TNF-α, IL-6 and IL-1β also exert an adverse effect on the vasculature by promoting insulin resistance and monocyte infiltration of the endothelium (118), resulting in chronic inflammation and endothelial damage. TNF-α is equally culpable in CVD development, as it regulates NF-κB activation in cardiomyocytes, which can cause both cardiomyopathy and heart failure by increasing inflammation and inducing myocyte atrophy (119). On the contrary, adiponectin expression, which has been shown to be reduced in women with GDM but significantly increased in PE, improves glucose and lipid metabolism and endothelial function (120, 121). The increase of adiponectin in pre-eclamptic women is believed to be related to a compensatory feedback mechanism in response to significant vascular damage (121). Adiponectin also exerts cardioprotective effects in vascular disease, via adiponectin receptor 1 and 2 activation, resulting in an increase in differentiation and survival of both endothelial and vascular smooth muscle cells while reducing the pro-inflammatory macrophage response (122). As such, circulating adiponectin levels have been proposed as biomarker for both coronary artery disease and myocardial infarction.

### Insulin Resistance and Altered Lipid Signaling

In the last decade there has been a significant increase in our understanding of the pathogenic role of adipose tissue inflammation in mediating the pathophysiology of atherosclerosis and vascular damage and the disruption of insulin signaling with resulting insulin resistance (123). The insulin resistant state, initiated by immune cell infiltration, metainflammation, and lipid signaling, impairs triglyceride storage resulting in increased FFA release. The increase in these FFAs is known to induce further insulin resistance in both the muscle and liver (124).

Insulin resistance, whether induced from immune cell infiltration in adipose tissue, subsequent pro-inflammatory responses or an increase in circulating FFAs, is considered an independent predictor of CV mortality and morbidity (125), in particular hypertension, type II diabetes and atherosclerosis (126, 127). The compensatory hyperinsulinemia associated with insulin resistance has been linked to vascular plaque formation in pathophysiology of atherosclerosis, induced by changes in gene expression of the estrogen receptor (128, 129). Insulin resistance-induced dyslipidemia is also postulated to be a significant risk factor for CVD, as it is characterized by increased circulating triglycerides, decreased HDLs as well as the reported formation of small dense low-density lipoproteins (sdLDLs) (128). Adipocytes play a key regulatory role in dyslipidemia. Impaired adipocyte function leads to a downregulation of FFA release suppression in response to insulin, as well as inducing hypertriglyceridemia and decreasing HDL concentrations (130), which can account for an increased CVD risk. This abnormal lipid metabolism is an early clinical marker for future maternal cardiometabolic disease and chronic endothelial dysfunction.

An increase in maternal adiposity and dysregulation has been correlated to impaired placental function through lipid accumulation, inflammation and oxidative stress (131). This increase in placental lipotoxicity is not resultant of increased lipid uptake from the maternal circulation, as fatty acid (FA) uptake has been shown to be decreased in cases of maternal obesity (132), but is the result of an increase in lipid esterification and storage and FA β-oxidation (FAO) dysregulation. A reduction of lipid transportation intermediates, specifically acylcarnitines, has been noted in the placentae of obese women (131, 133). These placentae also had higher mRNA and protein expression of FA esterification regulators such as peroxisome proliferator-activated receptor γ and acetyl-CoA carboxylase. Furthermore, extramitochondrial (peroxisomal) FAO is upregulated in the placenta of obese mothers, compensating for an impaired mitochondrial function. This pathway not only presents a rationale for localized placental endothelial dysfunction in both PE and GDM, but also that of enhanced ROS production and pro-inflammatory signaling through immune cell infiltration and mitochondrial dysfunction.

The detrimental effects of both insulin resistance and altered lipid signaling on endothelial function are believed to stem from endothelial-derived NO deficiency and ROS production (134). This relationship exists as a negative feedback loop where insulin resistance and metabolic cellular disturbances increase the production of ROS and reactive nitrogen species (RNS). As described earlier, these signals can deleteriously affect the function of eNOS and NO, rendering them incapable of their homeostatic vascular function. In turn, induced endothelial dysfunction impairs insulin action due to damage of microcirculatory blood flow and the capillary network, reducing the motility of insulin in the circulation. Vascular damage, resulting from lipid deposition and oxidative stress to the vessel wall, instigates an inflammatory response, and the release of chemoattractants and cytokines which further exaggerates insulin resistance and endothelial dysfunction (134). Adipokines such as retinol binding protein 4 (RBP4) can further detrimentally affect maternal glucose tolerance, by disrupting the activity of glucose transporter type 4 (GLUT4) (135), an insulin-sensitive glucose transporter that acts to promote insulin-stimulated glucose uptake by adipose, skeletal and cardiac tissues (136). RBP4 expression is increased in the omental adipose tissue of severely obese patients, suggesting that these proteins may be reciprocally connected in a glucose metabolism pathway (137). Similarly, TNF-α also reduces insulin-stimulated cellular glucose uptake by disrupting GLUT4 activation (138).

It has been proposed that the sphingolipid ceramide is also an important effector of insulin resistance through a signaling network linking lipid-induced inflammatory exacerbation to TLR-4 activation (106). TLR-4 dependent insulin resistance is activated by elevated endogenous and exogenous ligands such as FFAs and enteric lipopolysaccharides (139). TLR-4 activation results in activation of pro-inflammatory motifs c-Jun N-terminal kinases (JNK), IκB kinase (IKK), and the p38 mitogen-activated protein kinase which impair insulin signal transduction directly through inhibitory phosphorylation of insulin receptor substrate (IRS) on serine residues, preventing insulin-dependent glucose uptake (139). Activation of TLR-4 can also further stimulate pro-inflammatory cytokine signaling, leading to inhibition of glucose uptake as described previously.

### Advanced Glycation End Products

Advanced glycation end products (AGEs) are a heterogeneous group of compounds that are formed by non-enzymatic reactions between the carbonyl groups of reducing sugars and the free amino groups of proteins, lipids or nucleic acids. Elevated glucose levels resulting from maternal insulin resistance have the ability to bind to free amino groups of proteins, lipids or nucleic acids, modifying their function (140). Reducing sugars, such as glucose, can undergo enzymatic reaction to form these AGEs which can accumulate both intracellularly and extracellularly, disrupting the normal cellular function throughout the body (141). AGEs can also originate from exogenous sources such as dietary consumption and the majority of them are classified as toxic compounds which may stress cells and trigger cell injury, leading to pathological endothelial cell dysfunction (140).

Receptor for AGEs (RAGE) are cell surface pattern recognition receptors present in various cell types in the vasculature including endothelium, smooth muscle and macrophages (142).

As a result, AGEs have been implicated in mediating secondary outcomes of PE and GDM in part due to their detrimental effects on endothelial function (143, 144). Induced endothelial cell toxicity has also been linked to the progression of atherosclerosis and CVD and it has been suggested that therapeutics targeting AGEs may effectively ameliorate pathologies intrinsic to endothelial dysfunction (145, 146).

A recent study reported increased RAGE protein expression in PE placentae and that AGE formation, and the corresponding activation of RAGE, induced a trophoblastic inflammatory response in the placenta, increasing the secretion of pro-inflammatory cytokines. Soluble RAGE levels were also higher in serum in these pre-eclamptic mothers, postulating their role in modulating an inflammatory response in the maternal circulation and highlighting their potential deleterious effects on the maternal vasculature. Interestingly, RAGE mRNA transcription was comparable in PE women vs. women with uncomplicated pregnancies, suggesting that differential regulation of RAGE occurs post-transcriptionally (147).

A similar study found that AGE levels in maternal plasma in GDM were significantly higher than in women with uncomplicated pregnancies (148). The hyperglycemic state of GDM promotes AGE formation, a reaction which is further accelerated in a microenvironment of inflammation and oxidative stress (149). Additionally, it has been postulated that glycation of mitochondrial proteins alters their function, thereby disrupting mitochondrial bioenergetics with a resultant increase in ROS (150). Hemoglobin A1c (HbA1c), also known as glycosylated hemoglobin, has a significant diagnostic role in diabetic glycemic monitoring and has been demonstrated to correlate with arterial stiffness and endothelial dysfunction in patients with uncontrolled diabetes mellitus, particularly those with resistant hypertension. Moreno et al. found that, in these patients, HbA1c was an independent risk factor for impaired FMD and arterial stiffness, suggesting HbA1c as a useful biomarker for vasculopathy (151).

Circulating AGEs also represent a clinical risk in PE. To investigate the effect of these circulating pathogenic mediators in PE on adipose tissue, Akasaka et al. treated human primary adipocytes with PE serum. Significantly elevated mRNA expression of IL-6, C-C motif chemokine ligand 2 (CCL2) and RAGE were evident when compared with adipocytes treated with control serum. This suggests that upregulation of IL-6 and CCL2 expression is mediated in part by the RAGE pathway, resulting in the exacerbated pro-inflammatory response evident in PE (152).

### Soluble Fms-Like Tyrosine Kinase-1

Placental soluble fms-like tyrosine kinase-1 (sFlt-1), an antagonist of vascular endothelial growth factor (VEGF), is considered an etiological factor of endothelial damage in obstetric complications, in particular PE (153). PlGF, a member of the VEGF family, is a glycoprotein which regulates blood vessel development and maintenance of endothelial function (154). Whereas, VEGF can bind to various receptors including VEGFR-1, 2, and 3, PIGF specifically signals through VEGFR-1 (or Flt-1) binding (155). sFlt-1, the soluble variant of VEGFR-1, is an alternatively spliced variant of VEGFR-1 which inhibits angiogenesis by reducing free circulating levels of VEGF and PlGF through direct binding and inhibition of further interaction with receptors (156). sFlt-1 is released from the placenta in response to oxidative stress and inflammation (157) and the increased level of sFlt-1 is associated with detrimental alterations in endothelial integrity (158). sFlt-1 release from the placenta is also believed to be modulated by endogenous proteases, such as serine protease chymotrypsin-like protease/chymase and metalloprotease (159). The release of the anti-angiogenic factor sFlt1 presents a significant cause of proteinuria, glomerular endotheliosis and hypertension in obstetric complications through reduction of PlGF and VEGF bioavailability (160) and placental-derived sFlt-1 circulating concentrations are elevated in both PE and GDM pregnancies relative to healthy uncomplicated pregnancies (160, 161).

The significant correlation between PlGF inhibition by sFlt-1 and consequent pre-eclamptic vasculopathy presents a potential biomarker for the obstetric complication of PE. A recent cluster-randomized controlled trial found that the availability of serum PlGF level testing significantly reduced the time to clinical diagnosis and supported a lower incidence of maternal adverse events (162). These findings strongly endorse the use of circulating PlGF level testing as a biomarker and diagnostic test for PE.

Recent evidence has suggested that transcriptionally active syncytial aggregates, released from the placenta, act as a transporters for sFlt-1 in the maternal circulation (163). These syncytial knots are formed from abundant syncytiotrophoblast aggregation at the surface of placental terminal villi and are believed to be a marker of oxidative damage (164). These knots are enriched in sFlt-1 protein, which is heavily matrix-bound, and may be a novel transport mechanism for sFlt-1 and other damaging proteins in the maternal circulation (163).

Maternal obesity may affect these anti-angiogenic factors via a number of adipokine-mediated pathways (165) but their cross-talk is poorly characterized in human pregnancies. Interestingly, sFlt-1 was found to be secreted by adipose tissue explants obtained from non-pregnant human females with a robust inverse correlations between sFlt-1 expression and BMI (166). Contrary to this, no evidence supported sFlt-1 release of human adipocyte origin isolated from PE patients (167). Additionally, an increase in circulating sFlt1 levels strongly correlated with leptin concentration, but only in normal-weight pregnant women (168). These findings point toward a moderate, physiological production of anti-angiogenic proteins by visceral adipose tissue which is under autocrine regulation of adipokines and may be suppressed by insufficient placental signaling.

### Extracellular Vesicles

All eukaryotic cells are capable of secreting various types of membrane vesicles, known as extracellular vesicles (EVs), to carry or exchange a wide range of cargo involved in multiple physiological and pathological processes. In healthy pregnancy, these vesicles participate in many important physiological activities including embryo implantation, immune-modulation, spiral artery remodeling and metabolism adaptations (169).

EV-mediated crosstalk between the placenta, adipose tissue and circulating blood cells is of significant interest in relation to cardiovascular complications in pregnant women as circulating EV levels have been linked to both cardiovascular and diabetic complications in non-pregnant patients (170).

In terms of size and composition, various trophoblast-derived EVs are present in the maternal circulation from as early as 6 weeks of gestation (171, 172). Salomon et al. have shown that oxygen tension regulates the number and protein content of trophoblast EVs, with greater release under hypoxic conditions (173). Hyperglycemia also enhances the release and bioactivity of these vesicles (174). A great deal of *in vitro* and *in vivo* work has confirmed the immunomodulatory potential of placenta-derived EVs and their direct interaction with endothelial cells, monocytes, lymphocytes, neutrophils, macrophages and platelets (175–178).

A strong connection has been described between circulating trophoblast-derived EVs and pregnancy complications such as PE, IUGR and GDM (179–181). Syncytiotrophoblast clustering and abnormalities are hallmarks of placental insufficiency and pathology (182) and released EVs are linked to endothelial damage, monocyte stimulation and an upregulation of the maternal pro-inflammatory response (180). This phenomenon is also characteristic of GDM, where recent work suggested an alteration in miRNA content in trophoblast-derived EVs in the sera of GDM patients (183, 184). These particular miRNAs were proposed to act as important regulators of trophoblast differentiation as well as in insulin secretion and glucose transport in pregnant women (184) and therefore may be key regulators of GDM pathology.

Systemic maternal endothelial dysfunction often ensues in response to the circulating trophoblast-derived mediators (2). Firstly, epigenetic mechanisms can induce chronic changes in the endothelial function of both the mother and the fetus, instigated by miRNA release and resulting alterations of epigenetic machinery. *In vitro* data has demonstrated that in conditions such as GDM, which causes *in utero* environmental perturbations, endothelial functionality is decreased through increased miR-101 expression and reduced EZH2-β and trimethylation of histone H3 on lysine 27 levels (185). The direct role of miRNA trafficking by placenta-derived EVs may be a novel concept in the context of cardiovascular complications associated with pregnancy and it is yet unclear which cells/tissues are the predominant targets of this type of messaging.

Cronqvist et al. recently provided evidence for the EV-mediated transfer of PE and healthy placenta-specific miRNAs into the endoplasmic reticulum and mitochondria of primary human coronary artery endothelial cells (HCAEC) (186). This process resulted in a down regulation of specific target genes, including sFlt-1 in HCAEC, in response to both normal and PE EV-treatment (89), suggesting that EVs may be initiators rather than modifiers of the disease. In another similar study design, placenta-derived EVs from patients with PE reduced NO production and eNOS expression in primary human umbilical vein endothelial cells (HUVECs) (187). More recently, postpartum profiling of blood miRNA content identified a specific combination of miRNAs associated with higher cardiovascular risk in women with PE and hypertensive pregnancy due to endothelial dysfunction (188).

Adipose tissue-derived EVs have been traditionally isolated from culture medium of adipose tissue, adipocytes, and adipose tissue-derived stem cells (ADSC). The size and composition of these EVs are similar to their parent cell, and usually secrete various adipokines including adiponectin, leptin, resistin, and pro-inflammatory cytokines that can facilitate communication with immune cells (189). EVs are also enriched with enzymes, such as glucose-6-phosphate dehydrogenase, fatty acid synthase and lipids (190), indicating the possibility of these cellular mediators being primarily involved in the pathogenesis of GDM. Recent research investigating adipocytes-derived EVs, found that these inflammatory EVs upregulate VCAM-1 production in vascular endothelial cells which stimulates exaggerated leukocyte attachment, promoting inflammation and endothelial dysfunction (191). Further evidence from Kranendonk et al. established that specific markers of adipose tissue-derived EVs were associated with a 57% increased risk of developing metabolic syndrome and 16% increased risk of developing T2DM in patients already diagnosed with CV disease (192). This evidence outlines the complex but substantial impact that circulating EVs, derived from both placental and adipose tissue in cases of obstetric pathology, may have on maternal endothelial function and cardiometabolic health.

## Therapeutic Strategies

**Figure 2:**
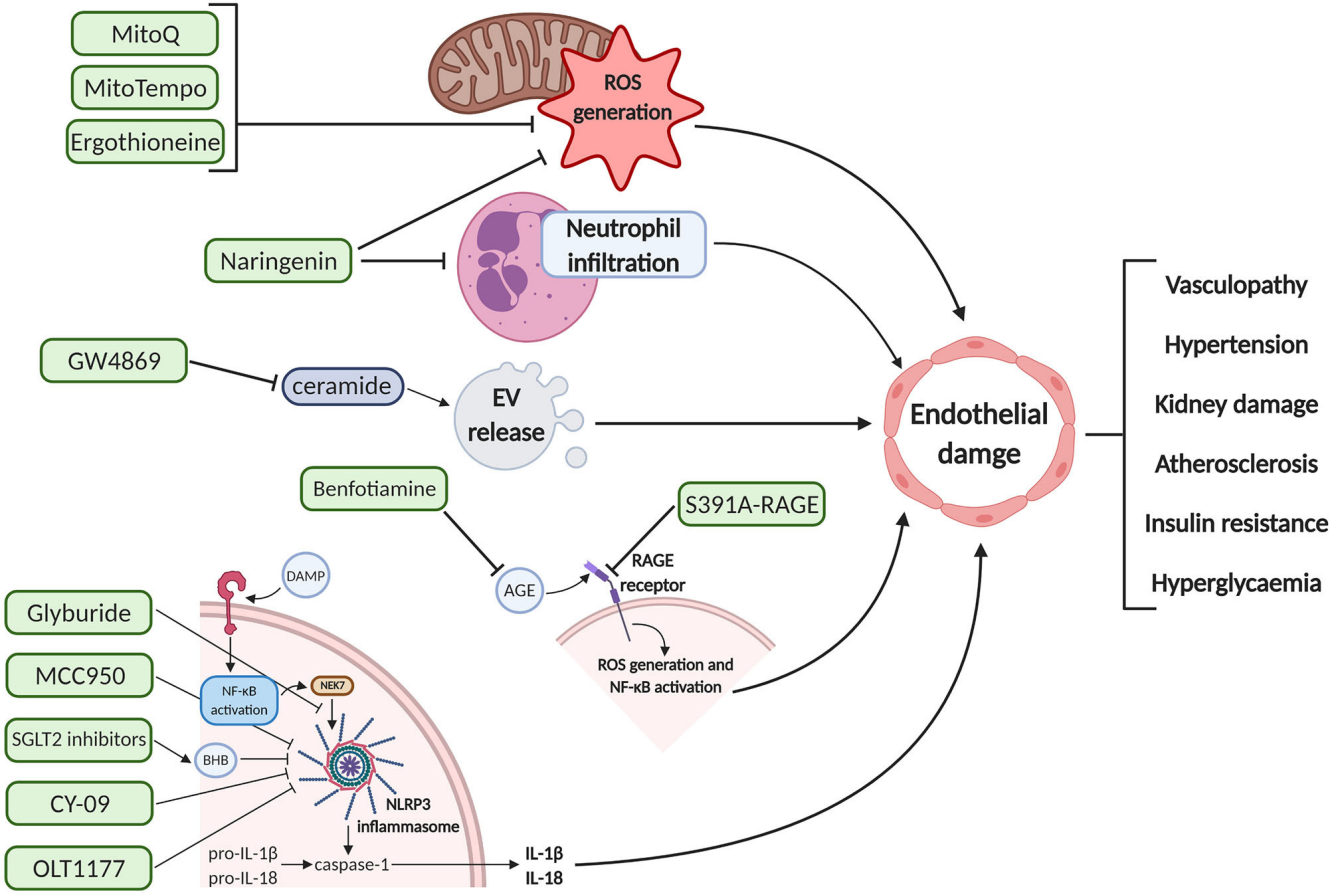
Schematic diagram of the pharmacodynamics of various proposed therapeutic interventions for endothelial damage prophylaxis. These agents can inhibit deleterious pathways upstream of endothelial cell dysfunction, potentially preventing the maternal clinical manifestations associated with pre-eclampsia and gestational diabetes mellitus. Created with BioRender.com. EV, extracellular vesicle; ROS, reactive oxygen species; DAMP, damage-associated molecular pattern; NF-κB, nuclear factor kappa-light-chain-enhancer of activated B cells; NEK7, NIMA related kinase 7; NLRP3, NOD-; LRR,- and pyrin domain-containing protein 3; BHB, beta-hydroxybutyrate; IL, interleukin; AGE, advanced glycation end products; RAGE, receptor for AGE.

Currently, therapeutic options for GDM focus on insulins and biguanides such as metformin, which are initiated once measures such as lifestyle modification fail to effectively achieve glycemic targets. In PE, therapeutic intervention commonly involves the use of β-blockers such as labetalol to control hypertension and pre-delivery anticonvulsive medication such as magnesium sulfate to prevent the onset of maternal eclampsia. Aspirin is also prescribed routinely for primary prevention of PE-related vascular dysfunction (193). As addressed previously, vascular complications of pregnancy are not limited to the duration of gestation and may induce chronic vascular and metabolic conditions such as CVD and T2DM (194). In this section, we will describe a number of potential alternative therapeutics which may protect against endothelial dysfunction in pregnancy, in part by negating the detrimental pathways of some of the pathogenic mediators characterized earlier, and may afford safe prophylaxis for high risk pregnant women, thereby minimizing acute endothelial dysfunction and improved long-term cardiometabolic prognosis (Figure 2).

Given the potential pathogenic role that dysfunctional mitochondria play in mediating endothelial damage, we propose that mitochondrial-targeted antioxidants may provide therapeutic benefit for mothers diagnosed with certain obstetric complications such as PE and GDM. MitoQ, a mitochondrial-targeted antioxidant, is a ubiquinone moiety linked to a lipophilic triphenylphosphonium (TPP) cation (195). MitoQ has ameliorated endothelial dysfunction and improved vascular function in recent animal and human studies (196, 197). Oral administration of MitoQ has established therapeutic benefit in rodent *in vivo* models of cardiac ischemia-reperfusion (IR) injury, sepsis, diabetic kidney damage and hypertension, respectively (198). Furthermore, a recent randomized controlled trial with MitoQ in healthy older adults aged between 60 and 79 years demonstrated that brachial artery FMD was 42% higher after MitoQ administration for 6 weeks, vs. placebo. The treatment arm also showed significantly reduced aortic stiffness and plasma oxidized LDL. The CLEAR trial, treating Hepatitis C patients with MitoQ, showed a decrease in circulating markers of liver damage and was the first report of mitochondrial-targeted antioxidant clinical efficacy and safety (199).

MitoTempo, a similar mitochondrial-targeted antioxidant, has shown promising *in vitro* results in the treatment of oxidative stress and endothelial dysfunction (200). We have previously shown that MitoTempo exerts a protective effect against ROS-induced cell damage of endothelium. MitoTempo was also effective in attenuating mitochondrial-specific ROS and inflammatory signals generated by PE plasma mediators in exposed endothelial cells (200). Furthermore, an *in vivo* study showed that MitoTempo reduced hypertension in two murine models of hypertension, namely, angiotensin II-induced hypertension and DOCA-salt hypertension, in part by decreasing vascular ROS concentrations and increasing NO production, with the consequent improvement in endothelial relaxation and vascular tone (201).

A nutraceutical mitochondria-targeted antioxidant, L-ergothioneine (ERG), has also demonstrated potential therapeutic effects, particularly in PE (202). ERG is a water-soluble amino acid derived from histidine, which accumulates preferentially in high oxidative stress organs through the action of a specific organic cation transporter novel type 1 (OCTN1) (203). This nutraceutical also has the therapeutic benefit of having a particularly long half-life of ~30 days and a well-established ability to cross both the blood brain barrier and the placenta (204, 205). In a reduced uterine perfusion pressure (RUPP) rat model of PE, ERG significantly reduced hypertension and rescued fetal growth restriction (206). ERG was also found to significantly reduce the levels of circulating sFlt-1 and the production of mitochondrial-specific ROS *in vivo*.

There remains a question over whether the increase in mitochondrial dysfunction and exaggerated ROS generation precedes or proceeds the disease state, as the physiological criteria required may often be present long before a clinical diagnosis is confirmed. However, these mitochondrial specific therapeutics show promising results in favor of targeted antioxidant therapy in obstetric complications of endothelial dysfunction, where other clinical interventions may be contraindicated. In addition, a clinical trial of chronic MitoQ administration in healthy older adults has established a favorable outcome in reducing markers of vasculopathy and inflammation (197).

Another potential therapeutic strategy to preserve endothelial function is to attenuate immune dysregulation and inflammation through inhibition of the NLRP3 inflammasome pathway. The NLRP3 inflammasome is activated by a diverse range of stimuli including products of mitochondrial dysfunction and ROS (207). NLRP3 pathway inhibitors are currently under investigation for a range of inflammatory conditions, including type II diabetes and atherosclerosis, through both indirect and direct pharmacological targeting. Glyburide, a sulfonylurea drug, is used for T2DM treatment in the US (208). Both *in vitro* and *in vivo* studies suggest that glyburide inhibits activation upstream of NLRP3 and more recently has been proposed to inhibit the NEK7-NLRP3 interaction (209, 210). Although glyburide is not approved for GDM treatment by the FDA, the American College of Obstetricians and Gynecologists (ACOG) recommended in 2013 that “when pharmacologic treatment of GDM is indicated, insulin and oral medications are equivalent in efficacy, and either can be an appropriate first-line therapy” (211). A similar sulfonylurea compound, MCC950, is considered one of the most selective and potent NLRP3 inhibitors (212). Unlike glyburide, MCC950 has been shown to act directly on the inflammasome. Some proposed mechanisms of actions of MCC950 include blocking IL-1β processing by caspase-1 or inhibition of ATP hydrolysis and NLRP3 assembly (213, 214). A recent study found that MCC950 significantly decreased IL-1β release and the activation of caspase-1 in colonic explants and macrophage cells isolated from the chronic colitis murine model (215). Furthermore, MCC950 was recently shown to reduce the cholesterol crystal-induced IL-1β response in human placental tissue explant (216).

As an alternative to sulfonylurea therapy, sodium glucose co-transporter 2 (SGLT2) inhibitors have significantly reduced T2DM-mediated vasculopathy in clinical trials (ClinicalTrials.gov Identifier: NCT02964572). SGLT2 inhibitors can stimulate an increase in circulating β-hydroxybutyrate (BHB) levels, which suppress NLRP3 inflammasome activation in macrophages. This trial in T2DM participants found that SGLT2 inhibition had a similar capacity to reduce glucose levels to sulfonylurea, but significantly decreased IL-1β secretion compared to baseline, unlike sulfonylurea (217). CY-09, a compound that selectively blocks NLRP3 by directly binding to the ATP-binding motif of NLRP3 NACHT domain, inhibiting inflammasome assembly and activation, has shown similar therapeutic effects in diabetic complications (218). *In vivo* experiments demonstrated that CY-09 reversed metabolic disorders in a diabetic mouse model through inhibition of NLRP3-dependent inflammation. These mice showed significantly higher insulin sensitivity and reduced weight gain compared to control. These results were not evident in NLRP3 knockout mice, suggesting that CY-09 alleviates diabetic symptoms specifically through NLRP3 inflammasome inhibition.

Currently undergoing clinical trials for treatment of degenerative arthritis, OLT1177 also acts directly on NLRP3 inflammasome to inhibit IL-1β and IL-6 secretion, thereby reducing neutrophil infiltration and joint swelling (219). OLT1177 demonstrates a promising safety profile after oral administration in phase I trials and was shown to be selective for NLRP3 inhibition over other inflammasomes in murine studies (212). Additionally, this compound has a long half-life with minimal toxicity in humans at various doses (220). Although these inhibitors are not currently investigated in pregnancy, inhibition of the NLRP3 inflammasome presents as a promising therapeutic avenue to alleviate both the acute and chronic inflammatory and metabolic symptoms of PE and GDM.

RAGE expression is significantly increased in women diagnosed with PE and GDM (143, 221). Hence, inhibiting RAGE activation may alleviate symptoms of ROS generation, pro-inflammatory cascades, and subsequent endothelial dysfunction. Aminoguanidine, benfotiamine, thiamine, and pyridoxamine have been clinically investigated to treat vascular complications of types 1 and 2 diabetes by inhibition of AGE formation (222), however the results were mixed. Aminoguanidine showed initial promising results but treatment was later ceased due to adverse events (223). Benfotiamine, however, significantly reduced serum levels of AGE as well as markers of endothelial dysfunction and oxidative stress in T2DM patients who ate a high AGE meal (224). The safety profile and clinical efficacy of these therapies remain to be fully evaluated in pregnancy.

Specific targeting of RAGE is also being investigated as a more direct means of therapeutic intervention. An example of such a compound is the mutant RAGE peptide S391A-RAGE, which acts to inhibit transactivation of RAGE (225). In an animal model of atherosclerosis, S391A-RAGE successfully diminished angiotensin II-dependent inflammation, endothelial dysfunction and atherogenesis (225). However, these novel RAGE peptides have not yet progressed past preclinical testing and require further investigation.

An alternative therapeutic option relates to the inhibition of the release and uptake of circulating EVs. This is an ongoing novel area of pharmacology research that may have exciting implications in many disease states. Inhibition of ceramide (a key player in endosomal sorting and EV biogenesis) with GW4869, a neutral sphingomyelinase inhibitor, is one current pharmacological approach under investigation (226, 227). Although these findings are not focused on disease modification in pregnancy, this research may open therapeutic doors into targeting pro-inflammatory syncytiotrophoblast-derived EVs or miRNA-mediated communication in the maternal circulation.

As the safety profile of potential therapeutic interventions is paramount in pregnancy, naturally occurring flavonoids such as naringenin may be of particular interest in PE and GDM. *In vitro* research has demonstrated that apigenin and naringenin, two types of flavones, diminish endothelial dysfunction (228, 229). These studies showed inhibition of ROS production, caspase-3 activity and phosphorylation of NF-κB in treated endothelial cells. In rodent models of GDM, naringenin exhibited antidiabetic properties, reducing blood glucose levels and improving renal function in part through a reduction in cellular apoptosis (230). Naringenin has also been shown to effectively reduce neutrophil infiltration and activation, indicating a vasculoprotective effect through amelioration of an overall pro-inflammatory phenotype (231).

Lifestyle modifications have often been the first line of intervention, particularly for managing the symptoms of GDM. Reduction of dietary glucose intake and careful glycemic monitoring can successfully manage insulin resistance, without the need for drug intervention, in less severe cases of GDM. Some studies, such as the ongoing EMERGE clinical trial (ClinicalTrials.gov Identifier: NCT02980276), are currently investigating the most effective way to implement lifestyle modifications in addition to metformin, to reduce the need for insulin and the occurrence of hyperglycemic episodes and maternal weight gain. In cases of PE, symptoms may often be more severe and medical intervention is often necessary along with lifestyle management. As such, extensive pre-clinical investigations into safe and effective therapeutics, with appropriate pre-clinical models, targeting the diverse pathogenic mediators of PE and GDM pathophysiology, is key for clinical progression.

## Conclusion

Placenta and adipose tissue-derived signaling motifs can contribute to a systemic maternal inflammatory response with subsequent endothelial dysfunction in PE and GDM pathology. Crosstalk between the metabolic organs involved can further propagate these detrimental outcomes in both obstetric complications. Uncontrolled metabolic dysfunction in the mother will also have negative implications on the developing fetus, as inflammatory markers such as IL-6 and excessive circulating glucose can cross the placenta and enter into the fetal circulation (232), having a direct link to offspring neuropsychological disorders and increased adiposity (233, 234).

The poor cardiometabolic outcomes associated with PE and GDM can be linked to metabolic dysfunction of both placental and adipose tissue. Conditions of poor placental perfusion, ischemia and oxidative stress induce an array of inimical signaling pathways, including pro-inflammatory cytokines, antiangiogenic factors, AGEs, EVs, and syncytial aggregates, which can have permanent deleterious consequences on endothelial function in the maternal vasculature. Dysregulated adipose tissue can amplify a pro-inflammatory state in response to infiltrating immune mediators, leading to the release of pro-inflammatory adipokines such as resistin, TNF-α, and IL-6. Oxidative stress responses in this metabolic tissue can also result in the release of an abundance of oxidized lipids and proteins into the maternal circulation, leading to endothelial damage which has been strongly linked to the pathogenesis of CV diseases such as atherosclerosis, heart failure, myocardial dysfunction and T2DM (235, 236). The resulting systemic damage will have both acute and chronic cardiometabolic health implications later in the maternal lifetime.

The interplay between the numerous pathogenic mediators described in this review opens avenues for new therapeutic possibilities. Nutraceutical antioxidants such as L-ergothioneine are particularly attractive as a result of their favorable safety profile (203). However, while certain pharmacotherapies such as L-ergothioneine and flavonoid supplementation have been deemed safe for use during pregnancy (230, 237), other options such as NLRP3 and RAGE inhibitors require further investigation to establish their potential effects on the developing fetus. Deciphering the complex pathways involved in PE and GDM will help further elucidate the pathophysiology of both obstetric complications, while also providing a window of opportunity to improve future maternal cardiometabolic health by developing clinically effective targeted therapeutics.

## Author Contributions

CJM is the primary author of the article manuscript. ET and FM contributed to the contents and design of the manuscript. CMM contributed to the contents and design of the manuscript and provided oversight throughout the writing of the manuscript. All authors contributed to the article and approved the submitted version.

## Conflict of Interest

The authors declare that the research was conducted in the absence of any commercial or financial relationships that could be construed as a potential conflict of interest.

## Abbreviations

PE: pre-eclampsia
GDM: gestational diabetes mellitus
IADPSG: The International Association of Diabetes and Pregnancy Study Groups
OGTT: oral glucose tolerance test
ISSHP: International Society for the study of Hypertension in Pregnancy
AKI: acute kidney injury
FMD: flow-mediated dilation
CVD: cardiovascular disease
HDL: high-density lipoprotein
BMI: body mass index
T2DM: type 2 diabetes mellitus
ROS: reactive oxygen species
DNA: deoxyribonucleic acid
IUGR: intrauterine growth restriction
uNK: uterine natural killer cells
EVC: extravillous cytotrophoblast
MMP: matrix metalloproteinase
IKK: IκB kinase
NF-κB: nuclear factor kappa B
ICAM-1: intracellular adhesion molecule 1
VCAM-1: vascular cell adhesion molecule 1
IL: interleukin
TNF-α: tumor necrosis factor α
NO: nitric oxide
eNOS: endothelial NO synthase
iNOS: inducible NO synthase
PlGF: placental growth factor
EV: extracellular vesicle
ONOO-: peroxynitrite
mtDNA: mitochondrial DNA
ETC: electron transport chain
Cf-mtDNA: cell-free mtDNA
DAMP: damage-associated molecular patterns
TLR: toll-like receptor
oxLDL: oxidized low-density lipoprotein
sFlt-1: soluble fms-like tyrosine kinase-1
sENG: soluble endoglin
FFA: free fatty acid
ATP: adenosine triphosphate
MAP: mitogen activated protein
VEGF: vascular endothelial growth factor
RBP4: retinol binding protein 4
GLUT4: glucose transporter type 4
JNK: c-Jun N-terminal kinases
IRS: insulin receptor substrate
RNS: reactive nitrogen species
FAO: fatty acid β-oxidation
sdLDL: small dense low-density lipoprotein
AGE: advanced glycation end product
RAGE: receptor for AGEs
miRNA: micro ribonucleic acid
CCL2: chemokine ligand 2
HbA1c: glycosylated hemoglobin
HCAEC: human coronary artery endothelial cell
HUVEC: human umbilical vein endothelial cell
ADSC: adipose tissue-derived stem cell
TPP: triphenylphosphonium
IR: ischemia-reperfusion
ERG: ergothioneine
OCTN1: organic cation transporter novel type 1
RUPP: reduced uterine perfusion pressure
NLRP3: NOD-, LRR- and pyrin domain-containing protein 3
NEK7: NIMA related kinase 7
SGLT2: sodium glucose co-transporter 2
BHB: β-hydroxybutyrate.
